# Pelvic Reconstruction With a Novel Three-Dimensional-Printed, Multimodality Imaging Based Endoprosthesis Following Enneking Type I + IV Resection

**DOI:** 10.3389/fonc.2021.629582

**Published:** 2021-04-13

**Authors:** Zeping Yu, Wenli Zhang, Xiang Fang, Chongqi Tu, Hong Duan

**Affiliations:** West China School of Medicine/West China Hospital, Sichuan University, Chengdu, China

**Keywords:** Sacroiliac joint, pelvic tumor, hemipelvic reconstruction, pedicle screw-rod system, 3D-printed endoprosthesis

## Abstract

**Background and Purpose:**

Pelvic tumor involving Type I + IV resections are technically challenging, along with various reconstructions methods presenting unsatisfactory outcomes and high complication rates. Since predominating studies preferred adopting pedicle screw-rod system (PRSS) to address this issue, we designed a novel three-dimensional-printed, multimodality imaging (3DMMI) based endoprosthesis with patient-specific instrument (PSI) assistance to facilitate the surgical reconstruction of pelvic tumor involving Enneking Type I + IV resection. We aimed to investigate the clinical effectiveness of this novel endoprosthesis and compare it with PRSS in Type I + IV reconstruction.

**Methods:**

We retrospective studied 28 patients for a median follow-up of 47 months (range, 10 to 128 months) in this study with either 3D-printed endoprosthesis reconstruction (n = 10) or PRSS reconstruction (n = 18) between January 2000 and December 2017. Preoperative 3DMMI technique was used for tumor evaluation, PSI design, virtual surgery, and endoprosthesis fabrication. Clinical, oncological outcomes, functional assessments, and complications were analyzed between the two groups.

**Results:**

Minor surgical trauma with mean operative duration of 251 ± 52.16 minutes (p = 0.034) and median intraoperative hemorrhage of 2000ml (range, 1600, 4000ml) (p = 0.032) was observed in endoprosthesis group. Wide margins were achieved in 9 patients of the endoprosthesis group compared with 10 in the PRSS group (p = 0.09). The 1993 version of the Musculoskeletal Tumor Society score (MSTS-93) was 23.9 ± 3.76 in endoprosthesis group, which was higher than PRSS group (p = 0.012). No statistical significance was found in relapse between two groups (p = 0.36). Complications were observed in two patients in endoprosthesis group compared with 12 patients in PRSS group (p = 0.046).

**Conclusion:**

The novel design of this 3D-printed endoprosthesis, together with 3DMMI and PSI assisted, is technically accessible with favorable clinical outcomes compared with PRSS. Further study is essential to identify its long-term outcomes.

## Introduction

Pelvic bone tumors invading sacroiliac joint are rare, and its reconstruction after wide resection still remains one of the most technically challenging procedures (1, 2). In contrast to the anatomical characteristics of extremities, pelvic tumors with occult symptoms are frequently not diagnosed until considerable tumor size (3, 4). Wide oncological resection combined with adjuvant therapies has shown its therapeutic advantage over hindquarter amputation in terms of expected survival, local tumor control, and quality of life (5, 6). With the advancements of multi-modality fusion imaging, adjuvant therapies, and three-dimensional printing technologies, various researchers have illustrated limb preservations are feasible for selected cases (1, 2, 4, 7–9). The main concern in reconstruction is to re-establish the continuity of the pelvic girdle and prevent the subsequent collapses or rotations of residual ilium under weight-bearing conditions and retain favorable limb functions in the long term. Though various surgical reconstructions after wide resection have been proposed, complications such as infection, implant loosening, breakage, and limb length discrepancy after limb-salvage procedures, staying at a high level, cannot be ignored (7, 10–12).

Some studies have adopted 3D-printed endoprosthesis, on the strength of a single or two image modalities, to accommodate the unique defect in the reconstruction of pelvis defect after wide resection (13, 14).However, solitary modality of image has its limitations in revealing complex structures because different modality images exhibit distinctive advantages and disadvantages for revealing different anatomical structures with various purposes (15). Surgeons rely on comprehensive analysis and clinical experience to integrate different and complex imaging information to form a stereoscopic image, which is subjective among individuals and may contribute to inaccurate resection and reconstruction. 3D-multimodality image (3DMMI) has fully exploited the strengths of different imaging technologies by integrating different modality images to a single visualized 3D model with both comprehensive informativeness and precision, which has been first applied in complex neurosurgery and has yielded satisfactory outcomes but less reported in orthopedics field (16–18). Since the application of 3DMMI in the pelvic girdle reconstruction, especially in type I+IV reconstruction, are rare. Studies either focus on PRSS reconstruction (1, 4) or 3D-printed endoprosthesis reconstruction for periacetabular region invading sacroiliac joint with a single modality of the image used (8, 10, 19). The purpose of this retrospective study was to present our experience that adopting the 3DMMI technique in reconstruction of type I+IV resection and describe the clinical outcomes, as well as complications compared with PRSS reconstruction.

## Patients and Methods

### Patients Eligibility and Demographics

This is a retrospective case-control study of patients who had undergone iliosacral resection (Enneking Type I + IV) with reconstruction in our institution between January 2000 and December 2017. Data on demographics, tumor evaluations, operative duration, intraoperative hemorrhage, functional and oncological outcomes, as well as postoperative complications were collected (**Table 1**). The inclusion criteria for eligible participants were summarized as follows: (1) bone neoplasms classified as Enneking Type I + IV; (2) discontinuity of posterior pelvic ring after surgical resection; (3) anticipated surgical margin with a width of 20 mm for malignancies and 10mm for benign tumors as preoperatively evaluated; and (4) without major neurovascular structures involved. Exclusion criteria: patients underwent previous surgery at other institutions, recurrent tumor, pelvic neoplasms classified as any other Enneking Type, as well as hematologic malignancies were excluded from the study. To minimize surgical technique heterogeneity, all the surgeries were performed by two senior surgeons (Dr. Hong Duan and Dr. Chongqi Tu) from a single subdivision in orthopedic department

**Table 1:** Demographics, clinical outcomes, and complications of the patients in this study.

Patient number	Gender	Age (years)	Diagnosis	Enneking stage	Reconstruction	Surgical margin	Intraoperative blood loss (ml)	Operation time (min)	Follow-up(month)	Oncological outcome		Limb discrepancy (cm)	MSTS-93	Complications
										Local recurrence	survival status			
1	Male	38	Osteosarcoma	IIB	3D-printed endoprosthesis	Wide	1800	150	10		DOD	2	15	
2	Male	52	Osteosarcoma	IIB	Pedicle rod-screw, cement	Wide	3100	330	28		DOD	2	19	Implant breakage
3	Male	34	Ewing sarcoma	IIB	3D-printed endoprosthesis	Wide	2000	280	29		NED	0	21	
4	Male	21	Osteosarcoma	IIB	Pedicle rod-screw	Wide	1600	290	53		NED	0	21	
5	Female	64	Osteosarcoma	IIB	Pedicle rod-screw	Intralesional	5400	420	48	Yes	DOD	0	16	
6	Female	39	Chondrosarcoma	IIB	3D-printed endoprosthesis	Wide	2600	280	22		DOD	0	28	
7	Male	32	Ewing sarcoma	IIB	3D-printed endoprosthesis	Wide	3200	260	46		NED	2	26	Delayed wound union, deep infection
8	Male	50	Giant cell tumor	3	Pedicle rod-screw, cement	Marginal	2800	320	58		AWD	1.5	20	Implant breakage, delayed wound union,
9	Female	19	Chondrosarcoma	IIB	Pedicle rod-screw	Wide-contaminated	4200	380	78	Yes	NED	4	21	Delayed wound union, implant loosening
10	Female	37	Aneurysmal bone cyst	3	Pedicle rod-screw, bone graft	Wide	1800	170	52		NED	2	21	
11	Female	43	Malignant giant cell tumor	IIB	Pedicle rod-screw, bone graft	Marginal	2800	340	128		NED	0	21	Delayed wound union, deep infection
12	Female	22	Osteosarcoma	IIB	Pedicle rod-screw, bone graft	Wide	1800	220	48		DOD	3.5	26	
13	Male	45	Metastatic renal cancer	III	3D-printed endoprosthesis	Wide	2200	250	28		NED	1	25	Delayed wound union
14	Female	26	Osteosarcoma	IIB	Pedicle rod-screw, bone cement	Wide-contaminated	3200	360	60		DOD	3	20	Implant breakage, deep infection
15	Male	34	Chondrosarcoma	IIB	3D-printed endoprosthesis	Wide	1600	220	26		NED	1	26	
16	Female	37	Chondrosarcoma	IIB	Pedicle rod-screw, cement	Wide	2200	270	50		NED	2.5	25	Implant loosening,
17	Male	35	Chondrosarcoma	IIB	Pedicle rod-screw, cement	Intralesional	7700	310	30	Yes	DOD	0	10	Delayed wound union, deep infection
18	Male	26	Osteosarcoma	IIB	Pedicle rod-screw, cement, bone graft	Wide	2600	360	100		AWD	2	18	Delayed wound union
19	Male	37	Osteosarcoma	IIB	Pedicle rod-screw, bone graft	Intralesional	4100	320	52	Yes	NED	0	21	Pulmonary embolism, delayed wound union
20	Female	57	Chondrosarcoma	IIB	Pedicle rod-screw, bone graft	Wide	2900	230	22	Yes	NED	2	26	
21	Female	24	Osteosarcoma	IIB	3D-printed endoprosthesis	Wide	2000	270	36		NED	0	23	Delayed wound union
22	Male	40	Chondrosarcoma	IIB	Pedicle rod-screw	Wide	3100	280	60		DOD	2.5	19	
23	Female	33	Chondrosarcoma	IIB	3D-printed endoprosthesis	Wide	1600	230	27		NED	0	25	
24	Male	58	Metastatic lung cancer	III	Pedicle rod-screw, cement	Intralesional	4300	430	28	Yes	DOD	3.5	20	
25	Female	29	Osteosarcoma	IIB	3D-printed endoprosthesis	Wide	1700	220	48		NED	0	27	
26	Male	52	Metastatic lung cancer	III	Pedicle rod-screw, bone graft	Wide	3600	290	10		NED	3	16	
27	Female	26	Osteosarcoma	IIB	Pedicle rod-screw, cement	Wide	2000	220	72		DOD	1	17	Implant loosening, deep infection
28	Male	19	Osteosarcoma	IIB	3D-printed endoprosthesis	Wide-contaminated	4000	350	29	Yes	AWD	0	23	Delayed wound union,

MSTS-93, 1993 version of Musculoskeletal Tumor Society function assessment score; DOD, died of disease; NED, no evidence of disease; AWD, alive with disease.

A total of 28 patients matching the study criteria were identified, of whom 18 patients received pedicle rod-screw system reconstruction (PRSS) and 10 patients were reconstructed with a novel 3D-printed endoprosthesis. There were six males and 4 females who underwent endoprosthesis reconstruction with a mean age of 32.7 years (range, 19 to 45 years). In the PRSS group, there were nine males and 9 females with a mean age of 39 years (range, 19 to 64 years). All patients diagnosed with osteosarcoma were administrated a two cycle neo-adjuvant chemotherapy (doxorubicin and cisplatin) before surgery, and another two-cycle protocol (vincristine, doxorubicin, ifosfamide, and etoposide) were applied in two patients with Ewing sarcoma. Three patients diagnosed with metastatic tumor received corresponding chemotherapy at the oncology department in our institution. Preoperative radiotherapy was not a routine procedure in our department and was only applied in patients with inadequate margin and recurrences after operation.

### Endoprosthesis Design With 3DMMI Technique

**Figure 1:**
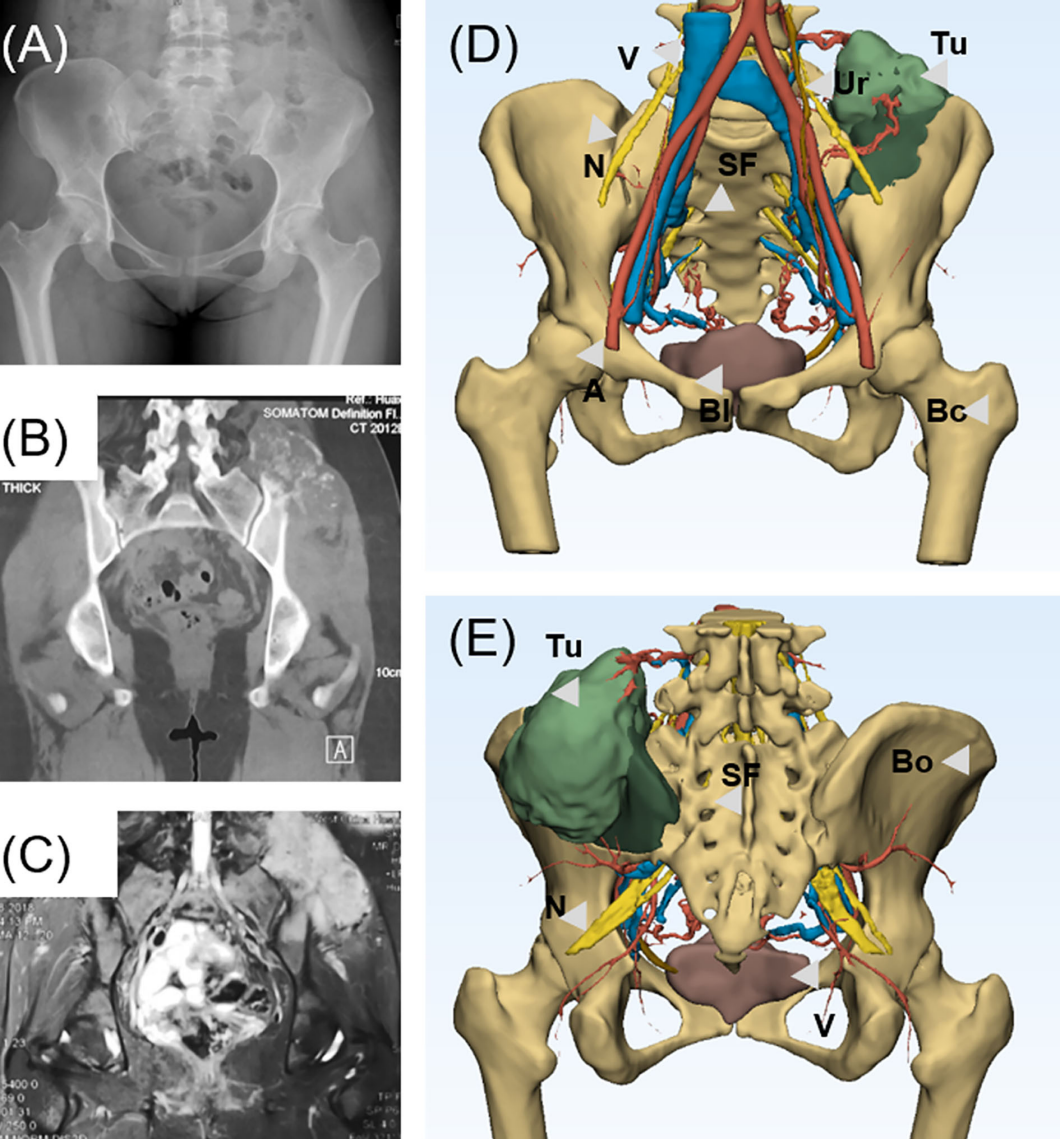
**(A)** Preoperative X-ray shows the bone destruction invading the left ilium and sacroiliac region. **(B)** Computed tomography shows the chondrogenic bone destruction in left sacroiliac region with soft tissue mass. **(C)** Magnetic resonance imaging shows an extensive soft tissue involvement invading sacroiliac region. **(D)** Anterior view of 3D-multimodality image of tumor model of various structures presented. **(E)** Posterior view of 3D-multimodality image of tumor model. 3DMMi, 3D-multimodality image; A, artery; Bl, bladder; Bo, bone; Bc, bone cortex; Ki, kidney; N, nerve; SF, sacral foramina; Tu, tumor; Ur, ureter; V, vein.

**Figure 2:**
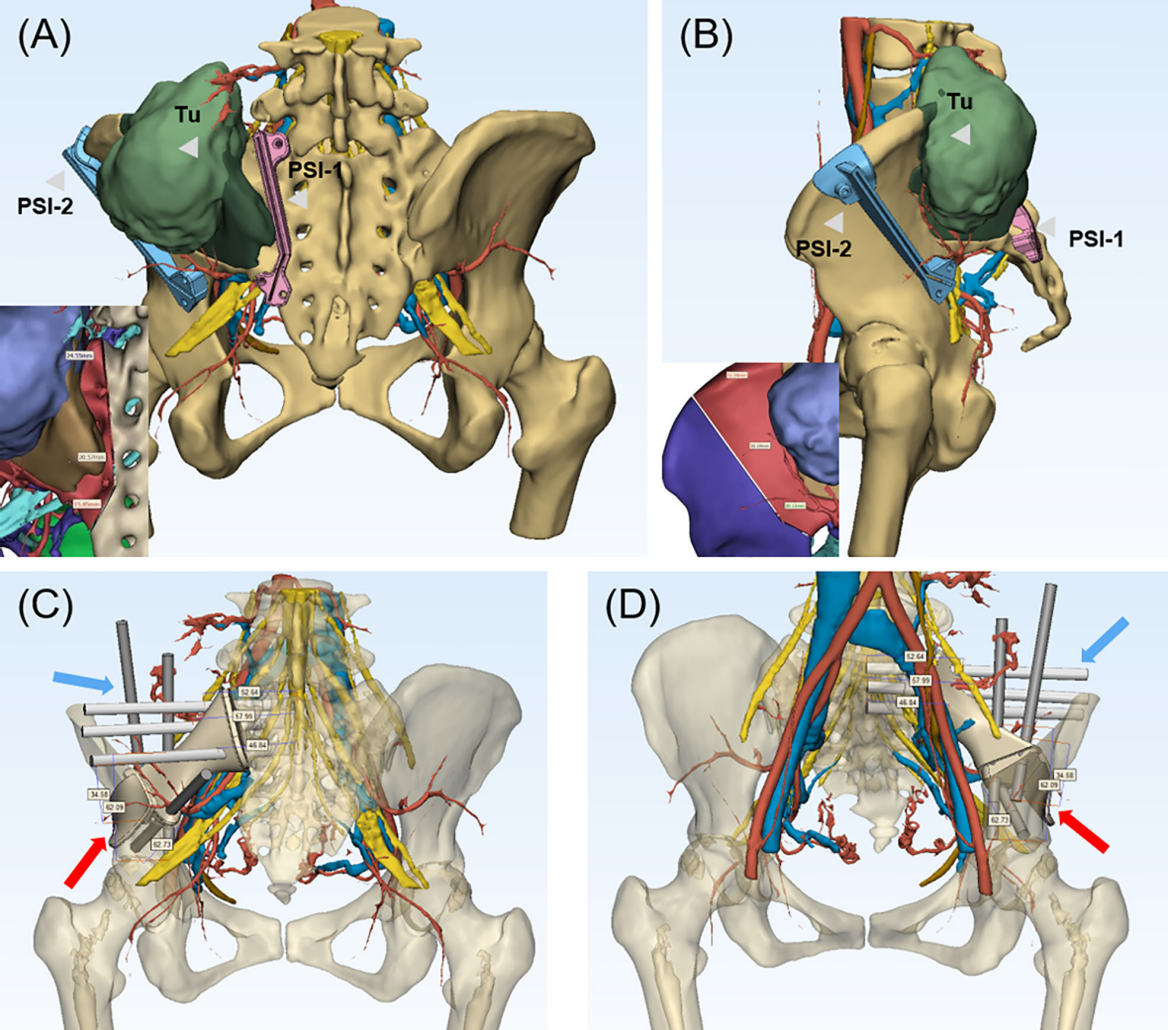
**(A, B)** 3D-multimodality image of tumor model with PSI and surgical margin designed. The PSI-1 was placed on the sacral ala adjacent to sacral foramina and PSI-2 was placed on ilium with a adequate margin designed. The neurovascular structure were clearly shown to avoid intraoperative damage. In the left bottom, the purple region represented tumor, and the brown region represented the tumor edema with details of neurovascular structures, which guaranteed the accuracy of the resection. **(A)** (Posterior view), **(B)** (Lateral view). **(C, D)** The endoprosthesis and screw fixation design with the length of screws shown on the 3DMMI of the patient after tumor resection. The screws in the sacral side was expected to reach the middle line of sacrum and the residual ilium was crossed fixed with the screws of the ilium side. The blue arrow indicated the screws and the red arrow indicated the endoprosthesis. PSI, patient-specific instrument; Tu, tumor.

In this study, a complete preoperative radiological examination was administrated for a detailed evaluation of the involvements and implementation of 3DMMI. Preoperative radiography assessments including a chest computed tomography (CT) and total-skeleton technetium-99 bone scanning were performed to confirm the distal metastasis sites of bone. X-ray was performed to evaluate the general condition of the bone and define whether a bone deformity exists. Contrast-enhanced CT (SOMATOM Emotion CT scanner) and magnetic resonance imaging (MRI) (Siemens Trio Tim 3.0T MRI scanner) of the pelvis were administrated to assess the extent of involvements in bony structures and soft tissue (**Figures 1A–C**). Vascular involvements were evaluated by computed tomography angiography (CTA), while neural involvements were evaluated by magnetic resonance water imaging (MRWI). MRI images were mapped to CT images through affine and diffeomorphic registration algorithm, which were all implemented in open-source software named Advanced Normalization Tools (ANTS). Bony structures and nerves were segmented from CT and MRI respectively using a level-set based segmentation algorithm implemented in open-source software ITK-SNAP (20). Quality assurance and manual correction were performed after automatic segmentation (accuracy of registration >95%, maximum segmentation error <2 mm compared with the raw DICOM data). The 3DMMI of tumor model based on 3DMMI technique was then accomplished (**Figures 1D, E**) and then exported to stereolithographic (STL) format and opened in a workstation running Reverse Engineering (RE) software Creo Parametric 2.0 (Parametric Technology Co., USA) to identify the characteristics for the resection margin and cut plane. In this study, the minimum tumor-free margin of 20 mm in bone were regarded as sufficient as indicated by a series of studies reporting the satisfactory tumor-free margin ranging from 5 to 15 mm and 20 mm for chondrosarcoma (21–23) and osteosarcoma with sensitive response to neoadjuvant chemotherapy (24). The cut plane was then designed with a minimum 20-mm margin to the tumor with comprehensive consideration of surgical approach, tumor-free requirement, avoidance of neurovascular injury and viscera organs, feasibility to the installation of the patient-specific instrument (PSI). Virtual PSI was added to the 3DMMI model once the cut plane was defined (**Figures 2A, B**).

**Figure 3:**
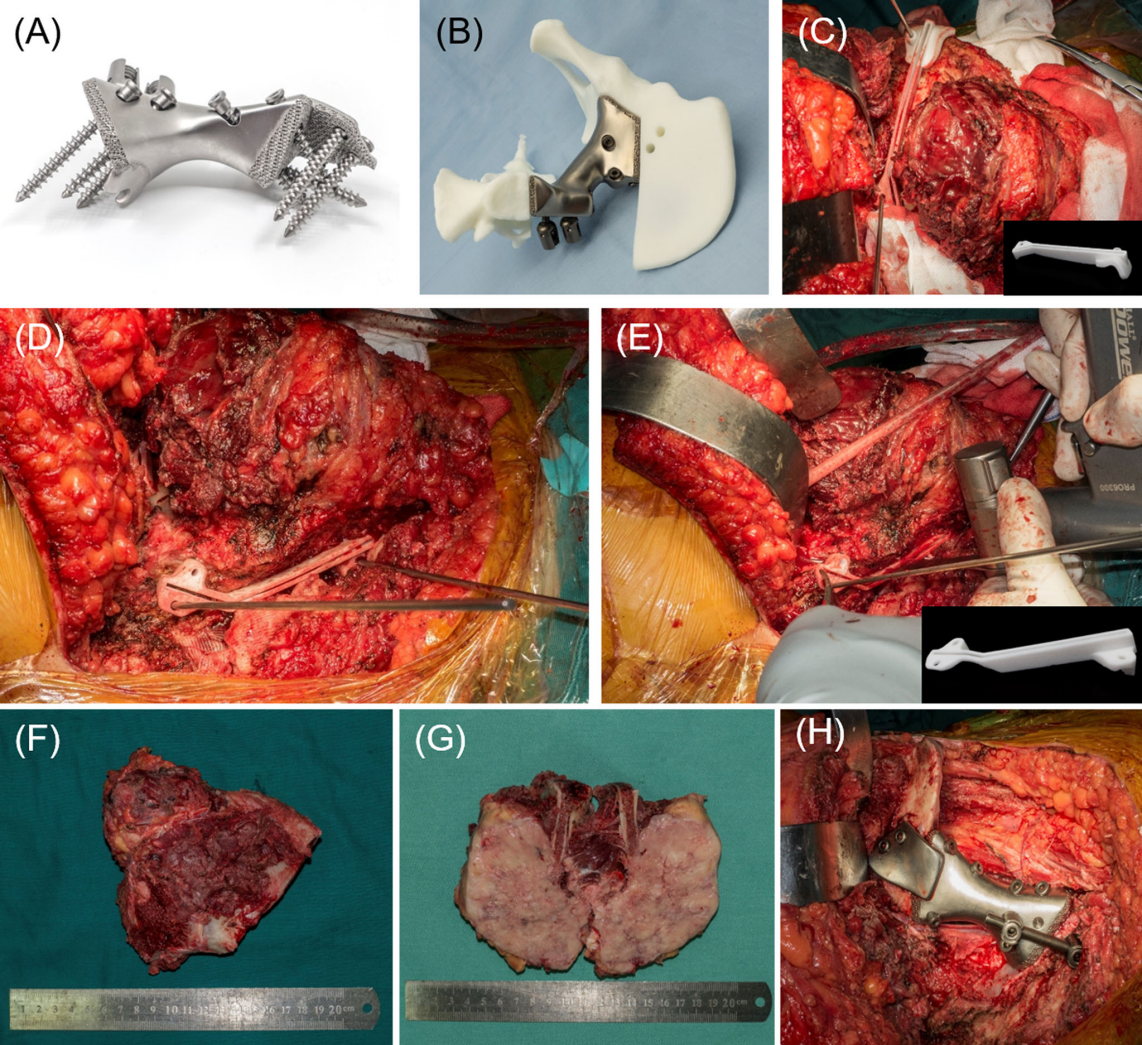
**(A)** The 3D-printed endoprosthesis with screws fixed on. Extra pedicle screw design was added in case of unsatisfactory implantation of the endoprosthesis. The contact interface of endoprosthesis-iliosacral region was modified as porous hydroxyapatite design to facilitate bone ingrowth and osseointegration. **(B)** The virtual surgery on printed model with 3D-printed endoprosthesis implantation. **(C)** The resection of the ilium side and tumor exposure with complete capsule. The PSI was fixed on the ilium with Kirschner wire. **(D)** The resection of the sacrum side. **(E)** The resection was done in a straightforward manner with swing saw through the groove designed in the PSI. **(F)** The resected tumor. **(G)** Section view of the resected tumor. **(H)** The endoprosthesis has been implanted precisely.

The endoprosthesis design was then designed to match the bony defect after resection, with several screw channels on the sacral lateral and iliac lateral. The length of cancellous bone screws was required to reach or exceed the cancellous bone screw for enhanced stabilization in implant-sacrum conjunction (**Figures 2C, D**). On the iliac side, conjunction was achieved through a T-shaped design with crossed screw fixation. Polyaxial screw was designed for extra stabilization and intraoperative adjustments in case of unexpected failure during the surgery. Furthermore, the polyaxial screw can be either fixed to the sacroiliac joint or designed to be a PRSS connecting vertebra and ilium. Finally, a rudimentary endoprosthesis (ChunLi Co, Beijing, China) was manufactured after streamlined modification for virtual operation on the printed tumor model (**Figures 3A, B**).

### Surgical Techniques

**Figure 4:**
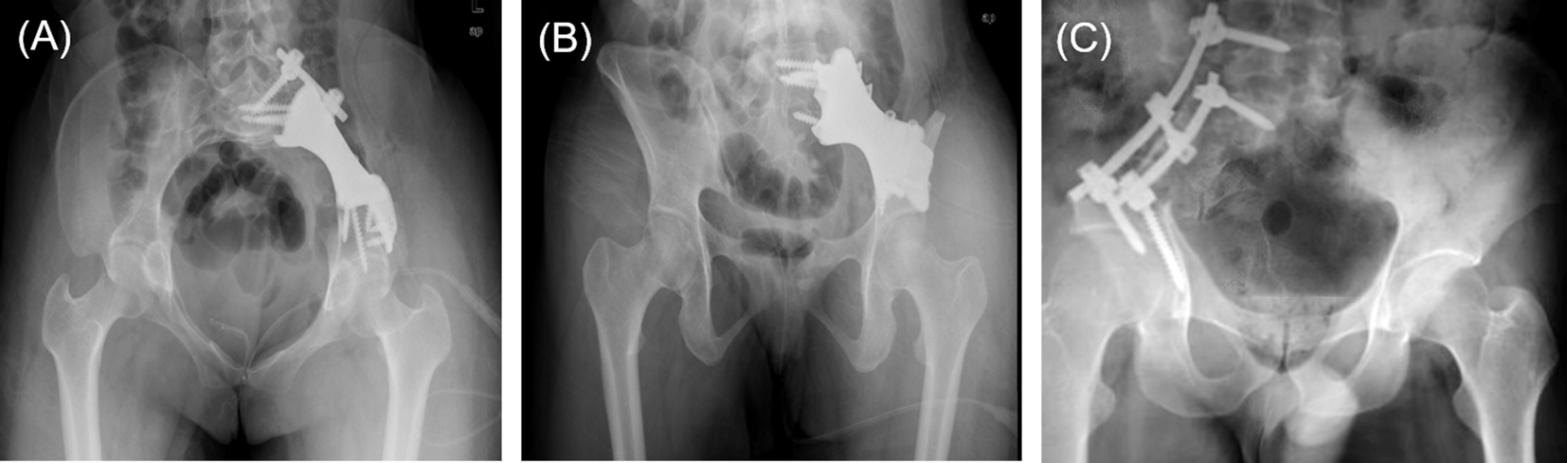
**(A, B)** Anteroposterior view of postoperative plain film of the patient underwent 3D-printed endoprosthesis. **(C)** An example of patient received pedicle screw-rod fixation.

The patients were placed in a lateral position on the contralateral side for patients who underwent endoprosthesis reconstruction. A posterior iliac incision extended with a posterior longitudinal midline approach was applied as previously reported in similar resections (25, 26). On the endopelvic side, abdominal muscles were detached from the iliac crest for exposure and protection of the external iliac vessels, femoral vessels, and nerves. On exoplevic side, gluteus muscles were detached from their origins to form a myocutaneous flap with superior gluteal neurovascular structures preserved. Subsequently, the dissection was performed on the endopelvic side down to the anterior cortex of the sacrum and identify the L4/L5 roots. Then, expose on the exopelvic side of ilium to the posterior aspect of sacrum. The exposure should be complete on previously located sites of PSI for the feasibility of PSI fixation and subsequent osteotomies. The PSIs were fixed with several 2-mm Kirschner wires (**Figures 3C–E**) and the osteotomies were performed in a straightforward manner using a swing saw on the sites of the ilium and sacral wing. If osteotomy was required to the sacral midline, osteotome would be applied on the anterior aspect. The tumor (**Figures 3F, G**) could be removed after dissection of sacrotuberous and sacrospinal ligaments. Since the endoprosthesis was a unique match to the defect, the reconstruction process, which has been described in the prosthesis design section, was carried on feasibly as preoperatively designed (**Figures 3H**, **4A, B**).

The reconstruction method in PRSS (Medtronic, Inc., USA) group was performed in the same surgical approach. Two pedicle screws were implanted in the lateral side of L5 vertebral bodies and sacrum while another two screws were placed in the supraacetabular regions. Two titanium rods were used to connect the screws (**Figure 4C**). Technological process of how 3DMMI, PSI and endoprosthesis were implemented in this study has been presented in **Video S1**.

### Postoperative Management

Customized lumbar pelvic hip braces were placed on all patients after surgery with the affected leg in a rotary neutral, 15° - 25°abduction, and 15°hip-flexion position. Three patients in the PRSS group presented with hemodynamic instability were admitted to the surgical intensive care unit (SICU) in our institution. The remaining patients were encouraged to early rehabilitation on the first day after surgery. Quadriceps isometric exercise and ankle flexion-extension exercises were executed during the first three days. Later on, active hip flexion not exceed 90° was executed to patents with a walking aid in the following two weeks. Partial weight-bearing was administrated since the third week and was gradually increased until normal weight-bearing status. The patients in the PRSS group started active hip flexion after 4 weeks and partial weight-bearing at 6 weeks timepoint. The adjuvant chemotherapy regimen was administrated in all patients with osteosarcoma and Ewing sarcoma. The follow-up regimen including physical examination and radiological tests were conducted monthly for the first three months and every 3 months thereafter in the first two years. After that, the follow-up was performed every 6 months. The function was evaluated by the Musculoskeletal Tumor Society (MSTS-93) score[13].

### Statistical Analysis

Continuous data with normal distribution were expressed as the mean ± standard deviation and data with non-normal distribution were expressed as median and range. Student’s t-test was used for continuous data with normal distribution, otherwise, Mann–Whitney U test was used. Overall survival (OS) was defined as death due to any cause. Disease-free survival (DFS) was defined as the time to relapse or death from any cause. Implant survival was defined as implant complication whether required revision or not. The OS, DFS and implant survival was analyzed with the Kaplan-Meier method. The log-rank test was used to compare the overall survival difference between the two groups. A p-value <0.05 was considered to indicate a statistically significant difference. Statistical analysis was performed with STATA 14.0 (StataCorp LLC., Texas, USA).

## Results

**Figure 5:**
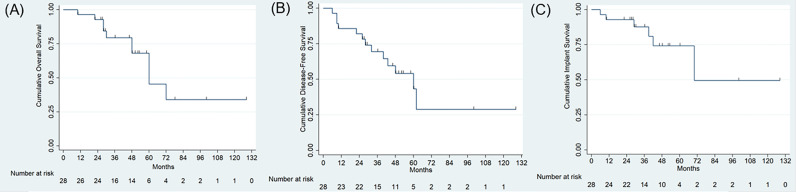
**(A, B)** Survival analysis using Kaplan-Meier curve showing the cumulative overall survival **(A)**, and disease-free survival **(B)** and implant survival **(C)** for all patients.

According to the Enneking and Dunham system (27), all patients were operated on a Type I/IV resection. Primary tumor origins and stage based on the Enneking staging system (28) were osteosarcomas in 12 patients (42.9%) with IIB, chondrosarcomas in 8 (25.8%) with IIB, Ewing sarcomas in 2 (7.1%) with IIB, giant cell tumor in 2 (7.1%) (malignant in one with IIB and benign in one with stage 3), and aneurysmal bone cyst in 1 (3.5%) with stage 3. Three patients diagnosed with metastatic cancers, which consist of pulmonary origin and renal cancer, were classified as stage III. The baseline data regarding age, gender, and adjuvant therapy in two groups showing no statistical significance. (p= 0.20, p=0.71 and p=0.68, respectively) (**Table 2**). The median follow-up in this study was 47 months (range, 10 to 128 months) for all patients and 28.5 months for the 3D-printed endoprosthesis group (range, 10 to 48 months) and 52 months for the PRSS group (range, 10 to 128 months). No patient lost follow-up by the time when data collected, or until the death of the patient. Adequate margins (wide margin) were biopsy-confirmed and were achieved in 9 (90.0%) patients in the endoprosthesis group and 10 patients (55.56%) in the PRSS group, which presented no statistical significance (p=0.09). We didn’t find significance regarding local recurrence rates between the endoprosthesis group (1/10, 10%) and PRSS group (6/18, 33.3%) (p=0.36). The cumulative OS was 96.4% (95% confidence interval, 77.2% to 99.5%), and 79.3% (95% confidence interval, 56.9% to 90.0%), 45.3% (95% confidence interval, 17.0% to 70.1%) at 1 year, 3 years, and 5 years, respectively for all patients. The DFS was 85.7% (95% confidence interval, 66.3% to 94.4%), and 69.5% (95% confidence interval, 47.9% to 83.6%), 43.3% (95% confidence interval, 18.7% to 66.0%) at 1 year, 3 years, and 5 years, respectively for all patients. Log-rank test for equality of overall survivor functions and disease-free survival showing no statistical significance (**Figures 5A, B**) (p = 0.68, p = 0.94, respectively).

**Table 2:** Comparison of baseline data, clinical outcomes, and complication between two groups.

	Pedicle rod-screw system	3D-printed endoprosthesis	*P*-value
Age	39.0 ± 14.0	32.70 ± 7.45	0.20
Gender			0.71^a^
Male	9	6	
Female	9	4	
Adjuvant therapy			
Chemotherapy	10	7	0.68^a^
Surgical margin			
Wide	10	9	0.09^a^
Wide-contaminated	2	1	
Marginal	2	0	
Intralesional	4	0	
MSTS-93^b^	19.83 ± 3.82	23.9 ± 3.76	0.012
Intraoperative hemorrhage	3,000 (1600, 7700)	2,000 (1600, 4000)	0.032^c^
Operation time	307.78 ± 70.0	251 ± 52.16	0.034
Limb discrepancy	2.5 (1, 4)	1.5 (1, 2)	0.03^c^
Recurrence			0.36
Yes	6	1	
No	12	9	
Complications^d^	12	2	0.046^a^
Deep infection	5	0	
Implant failures	6	0	
Wound-related	8	2	
Pulmonary embolism	1	0	

^a^χ2 test and Fisher exact probability method were used to compare the rate of wide resection and overall complication rate between two groups. ^b^MSTS-93, the 1993 version of the Musculoskeletal Tumor Society score. ^c^Mann-Whitney U test was used for the non-normal distribution of the data. The data was expressed as median and range. ^d^Deep infections occurred in 3 patients with delayed wound union. Implant failures occurred in 2 patients with deep infection.

We observed less intraoperative surgical trauma in patients who underwent 3D-printed endoprosthesis reconstruction evaluated by blood loss and operative time in this study. The median intraoperative hemorrhage was 3000 ml (range, 1600, 7700ml) in PRSS group, which was higher than 2000ml (range, 1600, 4000ml) in endoprosthesis group with statistical significance (p=0.032). The mean operation duration showing a statistically higher time cost of 307.78 ± 70.0 (range, 170, 430) minutes in PRSS reconstruction procedure compared with 251 ± 52.16 minutes (150, 350) in endoprosthesis group (p = 0.034).

The mean postoperative MSTS-93 score (%) at the last follow-up was 23.9 ± 3.76 (79.6%, range, 50% to 93.3%) in endoprosthesis group, compared with 19.83 ± 3.82 (66.1%, range, 33.3% to 86.7%) in PRSS group (p=0.012). Limb discrepancy was identified in four patients received endoprosthesis reconstruction and 12 in the PRSS group, which showed a significant difference of limb-length discrepancy between the endoprosthesis group (median=1.5 cm, range, 1 to 2 cm) and PRSS group (median=2.5 cm, range, 1 to 4 cm) (p=0.03). Seven of 18 patients in the PRSS group had more than 2 cm of discrepancy, while no patient with more than 2cm of discrepancy was observed in endoprosthesis group. The patient in 3DMMI group, with mildly abnormal gait, could walk and squat in a full range of motion freely without mobility assistive devices at the final follow-up (**Videos S2**) **and** (**S3**).

We observed 22 complications in 14 patients (50%), which presented as 20 complications in 12 patients in PRSS group compared with 2 patients in endoprosthesis group (p=0.046) (**Tables 1** and **2**). Wound-related problem (10/28, 35.7%) was the most frequent complication in this study. We treated these patients with dressing change, bacterial culture, and silver ion dressing (Atrauman Ag, Paul Hartmann Ag, German) and found no subsequent infection. One patient with pulmonary embolism was treated with anticoagulation therapy. Deep infection was found in 5 patients and was treated by debridement, intravenous antibiotics, and dressing change. The cumulative implant survival for PRSS group was 88.9% (95% confidence interval, 62.4% to 97.1%), and 82.1% (95% confidence interval, 53.7% to 93.9%), 67.1% (95% confidence interval, 37.5% to 85.1%) at 1 year, 3 years, and 5 years, respectively. Since no components related loosening, breakage, or implant-related revision was found in endoprosthesis group, we could not perform a log-rank test for comparison. The overall implant survival was shown in **Figure 5C**. Two patients (#14 and #27) with infection underwent curettage of the bony lesion in the debridement and were found subsequent implant-related complications, namely screw loosening and breakage. They presented chronic pain and walked with crutches and were treated with NSAIDs, and were closely followed without further surgical intervene. Another two patients (#2 and #8) were found screw breakages at the 24-month and 40-month follow-up. One (#2) of them was diagnosed with pulmonary metastasis with poor condition and refused further treatment. Another patient had screw breakage at residual ilium site with bursitis and received revision. Malposition of loosening rods occurred in Case 9 and Case 16 with restriction of activity and pain. They were all young patients and received revision surgery for functional demands.

## Discussion

Limb-sparing surgical procedure is much more challenging and demanding in the pelvis as compared with hindquarter amputation but has been proved to be associated with comparable life expectancies, relapse-free survival, and improved QOL (quality-of-life) (1, 7, 25). Due to the complex three-dimensional structures of the pelvis, especially when iliosacral regions involved, tumors are frequently related to the invasion of pelvic viscera and neurovascular structures and tend to develop into considerable sizes before confirmed, which render Type I + IV resection more difficult to achieve (1, 4, 7, 26). When the supra-acetabular region and the partial sacrum are resected, the discontinuity of the posterior pelvic girdle contributed to considerable limb shortening and decreased lumbopelvic stability if without reconstruction (19, 25). Nevertheless, few studies have presented reasonable outcomes in selected participants with no reconstruction (11, 12, 29). Currently, rare studies have unequivocally provided preferable functional outcomes with the known reconstruction methods (1, 7, 10, 25). The major concern is that practicable reconstructive modalities have not been proved to remain preferable functional outcomes and are related to various complications regarding mechanical failures and deep infections (7, 25, 30). To address the current issues, we adopted 3DMMI techniques with Patient-Specific Instruments (PSI) and customized 3D printed endoprosthesis, which allows surgeons to precisely resect the involved pelvis, restore integrity between the sacrum and the ilium and retain the acceptable functional outcomes.

Since the massive musculoskeletal resections are frequently mandatory for iliosacral tumors, it is still under debate whether the unstable pelvic girdle defects should be reconstructed (11, 12, 29). Beadel et al. (12) reported a retrospective case-control study with 16 patients who underwent Type I + IV resections involved, of which 12 patients received no reconstruction. In their study, shorter operative duration, minor hemorrhage, and fewer complications were observed in patients without reconstruction with functional evaluation showing no statistical difference in the two groups. In their updated retrospective observational study (29), 32 patients who underwent Type I or Type I/IV resections without reconstruction were enrolled with a mean MSTS-93 score of 67.3% and a 3% local recurrence rate. However, the complications were observed in 17 patients with 40.6% (13 of 32 patients) wound-related complications. They have achieved favorable oncological outcomes with adequate margins, which may indicate that more structures must be removed as they stated ‘aggressive resections’. If excessive resection without reconstruction, especially in the sacrum, the insufficient vascularized tissues with a large residual cavity may contribute to the infection (7). The main concept they prefer no reconstruction is that iliosacral defect induces the medialization of the hip joint center, accompanied with decreased body-weight moment exerting on a shorter abductor lever arm, which leads to an improved single-leg gait (12, 29). Nevertheless, the limb function is at the cost of the loss of lumbopelvic stability and integrity of pelvic ring. Besides, several studies have reported that sacroiliac arthrodesis contributes to progressive scoliosis and flail hips with considerable limb shortening (5, 7, 31). Wang et al. (32) reported a contrasting view that the functional outcomes of 12 patients with autograft and plate or pedicle screw fixation were superior to 12 cases without reconstruction, and the minor limb-length discrepancy was also defined in the reconstruction group. In the current study, we found no patient with more than 2cm limb discrepancy in endoprosthesis group, which leads us to believe that the lumbopelvic stability and pelvic girdle integrity should be restored with comparable results after Type I/IV resection.

The precision of resection with safe margins has a substantial impact on the oncological outcomes with regard to local relapse and patient survival. Peripheral regions of tumor are abundant with blood supply, which means resection process with positive margin is associated with a higher amount of hemorrhage and longer intraoperative duration. The results in our study have shown a minor surgical trauma in the prosthesis group with mean blood loss of 2270 ml (1600, 4000 ml) and mean operation time of 251 minutes (range, 150 to 350 minutes) which was comparable to the results of other studies ranging from 3153 to 5600ml and 256 minutes to 5.27 hours in similar resections regardless whether reconstruction performed or not (10–12, 26, 32, 33). Adequate surgical margins are particularly difficult to achieve when the sacroiliac joint is invaded (34) because substantial tumor size is frequently combined with extracompartmental feature, and occasionally responsible for distant tumor thrombi in the Baston plexus (31). Thus, 3D-printed endoprosthesis, characterized with individual design to fit the defects and improved accuracy of resection, is gaining growing popularities for precise resection purposes. However, most published literature presented their application of 3D-printed endoprosthesis based on one or two modalities of radiography with extremely limited structures merged into a 3D model (8, 13, 19, 35). Besides, the lack of PSI may lead to higher demands of surgical skills because inaccuracy resection wound make this technically difficult procedure even harder to perform (19). In this study, the cost for 3DMMI design was ¥3,000 to ¥5,000 and for PSI manufacture and 3D-printed endoprosthesis was ¥800 and ¥50,000, respectively, which is cost efficient when compared with the cost ranging from ¥50,000 to ¥60,000 in PRSS.

Although endoprosthesis group showed better outcomes than PRSS group regarding margin status, local relapse, we did not find a statistical difference between the two groups. The current study presenting an overall recurrence rate of 10% in the 3DMMI group with a wide margin rate of 90% is superior in resection of similar studies (**Table 3**). The reasons may be as follows. First, multiple modalities (CT, CTA, MRI, and MRWI) can be utilized and more details of the tumor can be presented, resulting in improved preoperative imaging assessments for the following resection and reconstruction. Unlike the most frequently used 3D printed endoprosthesis, designed and manufactured on the basis of a single modality of image mainly depends on bony structure (three-dimensional CT scan of the pelvis, design in Mimics), our 3DMMI technique implementing the algorithm in symmetric diffeomorphic and growcut manner, regionalization-adaptive registration, nD morphological contour interpolation, and Gaussian smoothing (18). Second, the application of PSI based on 3DMMI was precisely designed to be fixed in the tumor-free anatomic regions guarantees the reliability of the margin and accuracy. Due to the irregular shape of the pelvis and heterogeneity of the individuals, each PSI was unique and customized, which can only be fixed on the previously designated site. Third, the resection process was done in a straight-forward manner with time saved. PSI was firmly fixed on with K-wires and was designed with a groove. Osteotomy can easily be performed using a swing saw through the groove, which can prevent insufficient or excessive resection. Fourth, the preoperative virtual operation was performed, on the 3D-printed pelvis model with a customized endoprosthesis. Thus, we can test modify the design and operation plan to minimize the possible error and risk before surgery.

**Table 3:** A review of previous studies on sacroiliac resection and reconstruction, the margin, blood loss, proportion of patients with complications, and functional outcomes.

Study	Number of patients	Type of resection	Margin	Blood loss (mean, ml)	Reconstruction method	Follow-up(months)	Function (Mean)	Complications
Beadel et al. (12)	16 in total 12 (WR) 4 (R)	Type I for 16	Wide in 8 (50%) Microscopic or grossly positive in 8 (50%)	Mean 4325 for WR Mean 6250 for R	Four of 16 patients One (autograft + screw) Three (allograft + screw + cement)	Mean 45 for WR Mean 43 for R	Mean MSTS-93^c^ 58% for WR (n = 12) 51% for R (n = 4)	Overall complication rate: NR intraoperative vascular injury in 1 (6.3%, n = 16) Allograft fractured in 1 (6.3%, n = 16) Allograft nonunion in 2 (12.5%, n = 16) Spinal deformity in 1 (8.3%, n = 12) Wound infection or necrosis in 7 (58.3%, n = 12)
Gupta et al. (29)	32	Type I in 8 Type I/IV in 24	R0 in 21 (65.6%) R1 in 8 (25%) R2 in 3 (9.4%)	NR	WR for all patients	159	Mean MSTS-93 67.3%	Overall 53.1% (17 of 32 patients) Wound- or infection-related complication in 13 (40.6%, n = 32) Postoperative fracture in 2 (6.3%, n = 32) Local recurrence in 1 (3%, n = 32)
Lin et al. (1)	30	Type I in 24 Type I/IV in 6	NR	NR	PRSS^4^ ± bone cement ± bone graft	Mean 40.4(13.1 to 162.2)	Overall MSTS 81.0% (n=30)	Overall 40% (12 of 30 patients) Wound complications in 4 (13.3%, n = 30) Neurologic defects in 4 (13.3% n = 30) Bone nonunion in 1 (3.3% n = 30) mechanical failures in 5 (16.6% n = 30) (implant breakage in 1 and lessening in 4) Local recurrence in 10 (33.3%)
Jin et al. (11)	21	Type I/IV	Wide in 9 (42.9%) Marginal in 5 (23.8%) Intralesional in 7 (33.3%)	Mean 1988 for WR (n=18) Mean 3266 for R (n=3)	No reconstruction in 18 Autograft + screw in 3	Mean 67.3 (14 to 163)	Mean MSTS-93 93.3% for WR and R	Overall complication rate: NR Autograft absorption 1 (33.3%, n=3) Intraoperative sacral nerve damage in 1(4.8%, n = 21) Dislocation in 1 (4.8%, n = 21) Wound necrosis in 2 (9.6%, n = 21) Screw loosening in 1 (4.8%, n = 21) Local recurrence 16.7%
Wang et al. (10)	25	Type I+II+IV	Wide in 12 (48%) Marginal in 6 (24%) Intralesinal in 7 (28%)	Mean 5600	Combined hemipelvic endoprosthesis (PRSS)	Median 48 (23 to 87)	Mean MSTS-93 48.0% (range, 30.0–66.7%)	Overall 56% (14 of 25 patients) Wound disturbances in 7 (28.0%, n = 25 patients) Deep infections in 4 patients (16.0%, n = 25) Prosthesis-related complication in 7 (24.0%, n = 25) Intraoperative sacral nerve damage in 2 (80%, n = 25) Local recurrence in 8 (32.0%, n = 25)
Court et al. (7)	40	Type I/IV in 33 Type I/II/IV in 4 Type I/II/III/IV in 3	Wide in 20 (52.6%) Marginal in 8 (21.1%) Wide-contaminated 4 (10.5%) Marginal-contaminated 2 of 38 (5.3%) Intralesional in 4 (10.5%)	NR	PRSS in 21 Various pseudarthrosis/arthrodesis ± osteosynthesis in 19	Mean 70 (12-180)	Overall mean MSTS: NA 20% to 80% in different subgroups	Deep infection in 15 (37.5%, n = 40) Wound necrosis in 1 (2.5%, n = 40) Implant loosening in 5 (12.5%, n =40) Lumbosacral destabilization in 2 (5%, n = 40) Local recurrence in 9 (22.5%, n = 40)
Zang et al. (25)	17	Type I/II/IV in 16 Type I/II/III/IV in 1	Wide in 9 (52.9%) Intralesional in 4 ()	3153 (1700 to 6000)	Combined hemipelvic endoprosthesis (Combined with PRSS)	Mean 33 (15 to 59)	Mean MSTS 58% (33 to 77)	Overall 47.1% (8/17) Deep infection in 2 (11.8%, n = 17) wound healing in 5 (29.4%, n = 17) Dislocation in 1 (5.9%, n = 17) Local recurrence in 6 (35.3%, n = 17)
Zhang et al. (19)	20	Type I/II/IV in 9 Type I/II/III/IV in 6	Wide in 8 (40%) Marginal in 10 (50%) Intralesional in 2 (10%)	NR	Combined hemipelvic endoprosthesis (Combined with PRSS)	Median 36 (6 to 60)	Median MSTS 19 (5 to 26) for patients with 12 months survival or more (n = 16)	Overall in 12 (60%, n=20) Deep infection in 1 (5%, n=20) Dislocation in 2 (10%, n=20) Local recurrence in 3 (15%, n=20)
Ozaki et al. (30)	12	Type I/II/IV in 8 Type II/III in 4	Wide in 7 (58.3%) Marginal in 5 (41.7%)	NR	Custom-made hemipelvic endoprosthesis	Median 57 (26 to 77)	Overall mean MSTS 33.3% for patients that were recorded (n=8) Mean MSTS 37% for patients with prosthesis (n = NR)	Deep infection in 4 (33.3%, n = 12) Dislocation in 1 (8.3%, n = 12) Screw loosening/breakage in 2 (12.6%, n = 12) Local recurrence in 5 (41.6%, n = 12)
Wang et al. (32)	26	Type I/IV	Wide in 19 Marginal in 7	Mean 1,300 (range, 600–3,600) The mean operative time was 280 (range, 230–380) min	Fibular grafts and plate and/or PRSS	Median 84.4 (32 to 165)	MSTS WR: 20.36 ± 2.56 R: 25.25 ± 2.93	Overall 26.9% (7/26) Delayed wound healing in 1 (3.8%, n = 26 Screw breakage in 1 (3.8%, n = 26
Nassif et al. (34)	6	Type I/IV	wide in 3 marginal in 3	median of 256 minutes (range, 212–339 minutes). The Median reported blood loss was1400 cc (range, 900–6000 cc)	Autogenous iliac graft with PRSS	median 33(6 to 53)	Mean MSTS ‘93 score was 72%	Overall 67.7% (4 of 6 patients) Delayed wound healing in 2 (33.3%, n = 6) Deep infection in 1 (16.7%, n = 6) Implant failure in 1 (16.7%, n = 6)
Sabourin et al. (26)	24	Type I in 13 Type I/IV in 4 SI in 7	Marginal in 11 Wide in 12 Contaminated in 1	Average 5.27 hours (2.5—12).	Autogenous graft with pedicle s r	Mean 57.6 (4 to 240)	Mean MSTS-93 61.1%	Overall 75% (18/24) Deep infections in 8 (33.3%, n = 24) Hematomas in 6 (25%, n = 24) Scar necroses in 8 (33.3%, n = 24) Graft nonunion in 42% (n = NR)
Gebert et al. (45)	35	Type I/IV	Wide in 32 Marginal in 3	NR	PRSS with bonce cement in 29 PRSS in 6	Mean 46 (1.9 to 139.5)	Mean MSTS 21.2 (10 to 27).	Delayed wound healing in 8 (22.9%, n = 35) Deep infections in 5 (14%, n = 35) Implant failure in 6 (17.1%, n = 35) Neurological defect in 12 (34%, n = 35) Local recurrence in 1 (2.9%, n = 35)

MSTS, Musculoskeletal Tumor Society score; WR, patients without reconstruction; R, patients with reconstruction; NR, not report, PRSS, pedicle rod-screw system.

Among the existing reconstruction methods, we prefer to adopt endoprosthesis reconstruction of pelvic girdle for its mechanical superiority of transferring weight-bearing force from the lumbar spine to hip and limb *via* sacroiliac joint. In this study, the conjunction between the prosthesis and residual sacrum was enhanced by enlarged implant-sacrum contact surface and more screw fixation compared with similar studies (8, 19, 25). The contact of implant-sacrum was anatomic match with three screws perpendicularly fixed and one extra rod-screw fixation perpendicular to sacroiliac joint, while the interface of implant-ilium was strengthened by an ‘L’ shaped design with crossed screws fixed. The polyaxial screw design can be either directly fixed or adjusted for a rod-screw fixation purpose. The advantage of this design lies in transferring the shear stress to compression stress in the stress conduction (35). Pedicle screw and rod systems were favored predominantly in many studies. In this type of reconstruction, stress mainly concentrated on both ends of the connecting rods in the supraacetabular region, and on the pedicle bodies of L4/L5 or residual sacrum, which is vulnerable to torsional force (36, 37). Hence, some studies recommended a compound rod and screw system with bone grafts for improved lumbopelvic stability (1, 19, 32, 37). However, problems remain for bone healing, graft fracture, and infections (1, 7, 26, 32). Allografts have been widely recognized with high infection risks (1, 30), whereas autografts are limited by donor site morbidities and extra operations (1, 33). Despite the superiorities of autograft, Sabourin et al. reported poor functional outcomes and a graft nonunion rate of 42% using polyaxial screws and titanium rods in 24 patients who underwent autogenous graft with pedicle screw-rod system fixation (26).

Endoprosthesis reconstructions presented various functional performance according to types of resection and reconstruction, which include allograft/prosthesis composite (38), modular hemipelvic endoprosthesis (39, 40), modular saddle prosthesis (41), and custom-made hemipelvic endoprosthesis (19, 25, 42) (**Table 3**). Combined pedicle-hemipelvic endoprosthesis, the most frequently applied method in treating pelvis tumors invading sacroiliac joint, of which the mean MSTS score ranging from 48% to 63% (8, 10, 19, 25). These prostheses were pedicle-hemipelvic design with connecting rods fixed on the acetabular components and L4/L5 vertebra (8, 10, 25). The similar problems confronting the connecting rods with peak prosthetic stress at the conjunction regions (10, 37), as Wang et al. highlighted in their continuous studies (8, 10). Zhang et al. reported high complications concerning pedicle screw-rod failures, and prosthetic dislocation in their previous study (25) and improve the prosthesis design with enhanced stabilization by extra fixation to the residual sacrum (19). These pedicle-hemipelvic prostheses emphasized the fixation should be embedded in bone cement, thus enhanced stabilization can be retained. However, bone cement is well compression-resistant but vulnerable to torsion, as proved by the high failure rates of screw-rod with bone cement enhancement (19). Since studies specifically focused on this anatomic region are rare, it is difficult to make between-approach comparisons with other endoprosthesis. However, our endoprosthesis showing a mean MSTS-93 score of 23.9 (79.7%) was favorable in the Type I + IV resections. Apart from the mechanical limitations of stress concentration of connecting rods and screws, the sacroiliac joint is characterized with physiological micromotion and hip movements induce rotation in the supraacetabular region (43). This type of enhancements may yield initial stability rather than long-term benefits. Our implant failure of 33.3% in PRSS group may also support the above statements. The fixation of rod-screw on L5 or L4/5 may be another potential risk for decreased implant stability. Lin et al. conducted a study with various types of pedicle rod-screw fixations and found that extrapelvic fixation in L4/5 was less stable than intrapelvic fixation in residual sacrum (1). The study recommends intrapelvic fixation if possible, because micromotions between intervertebral discs may contribute to increased stress level (8) and further fatigue broken or loosening of implant (1).

Other complications that are mainly focused on wound-related disturbances and deep infections were the most frequent complications in this study, as widely recognized (7, 25, 29, 35). We found two delayed unions (20%) and no deep infection in the endoprosthesis group, compared with 44.4% and 27.8% in PRSS group. Apart from inherent potential risks that sacrum adjacent to rectum, presacral venous hemorrhage, and undermined tissue regeneration caused by neoadjuvant therapy, the prolonged operative duration, increased blood loss and insufficient soft tissue coverage may also contribute to the infection and wound union (9, 44). Minor surgical trauma is difficult to achieve by conventional freehand surgical techniques that reported deep infection rate ranging from 11.8% to 37.5% (7, 10, 25, 26, 33, 34, 45). However, we minimized the chances of infection in a few aspects. On the one hand, the size and shape of endoprosthesis volume are not necessary to be the same as the bone defect. Decreased volume design of endoprosthesis that maintain the biomechanics contributes to better soft-tissue coverage. On the other hand, PSI based on 3DMMI allows the surgeon to perform planned resections, which diminish unnecessary surgical manipulations (9, 14, 46). Hence, we think patients may benefit from a well-designed implant with 3DMMI-based preoperative evaluation, which yields decreased infection in this study.

Though we presented encouraging clinical outcomes in patients with endoprosthesis reconstruction in Type I/IV resections, this study had a few limitations. First, the present study was retrospective in nature and was limited by its small sample enrolled. However, studies focused on Type I/IV resection are few (4) and mainly included Type I resection without a control group. Hence, the clinical outcomes may be comparable and able to justify the application of the novel prosthesis design. Second, the follow-up was not long enough to detect major complications, especially implant-related complications in the endoprosthesis group. Since the contact surface of endoprosthesis was highly porous surface design mimicking bone trabecula structure, we think a mean follow-up of 30.1 months (endoprosthesis group) in a group with a limited lifespan may be sufficient to report its early implant function, as the similar design has proved its long-term stability (19, 44). Third, tumor heterogeneity exists in this study. Despite we included benign tumors, the main purpose of this study was to evaluate the performance of resection and reconstruction adopting this novel design with 3DMMI and PSI. And the baseline data were comparable between groups. To date, this may be the first study reporting specific Type I/IV resections reconstructed with 3D-printed endoprosthesis assisted by 3DMMI and PSI.

In conclusion, the novel design of this 3D-printed endoprosthesis, together with 3DMMI and PSI assisted, are technically accessible with relatively minor trauma and better implant stability in facilitating Type I/IV resections compared with PRSS. It must be noted that long-term follow-up is essential to validate its capacity.

## Data Availability Statement

The original contributions presented in the study are included in the article/**Supplementary Material**. Further inquiries can be directed to the corresponding author.

## Ethics Statement

The studies involving human participants were reviewed and approved by Medical Ethics Committee of West China Hospital, Sichuan University. The patients/participants provided their written informed consent to participate in this study. Written informed consent was obtained from the individual(s) for the publication of any potentially identifiable images or data included in this article.

## Author Contributions

Study Design: HD. Data Collection: ZY and WZ. Methodology: ZY, XF, and CT. Written Work: ZY and WZ. Review and Revision: HD and WZ. Visualization: HD and WZ. Resource: HD and CT. Project Administration: HD and CT. All authors contributed to the article and approved the submitted version.

## Conflict of Interest

The authors declare that the research was conducted in the absence of any commercial or financial relationships that could be construed as a potential conflict of interest.

## Supplementary Material

Supplementary Video S1 The video shows the flow in the current study including how three-dimensional multimodality imaging (3DMMI) works and the implantation of 3D-printed endoprosthesis.

Supplementary Videos S2 and S3 The patient, with mildly abnormal gait, could walk and squat in a full range of motion freely without mobility assistive devices at the final follow-up.
